# Phytoremediation effect of *Medicago sativa* colonized by *Piriformospora indica* in the phenanthrene and cadmium co-contaminated soil

**DOI:** 10.1186/s12896-020-00613-2

**Published:** 2020-04-28

**Authors:** Liang Li, Pengyue Zhu, Xiaoyang Wang, Zhenhua Zhang

**Affiliations:** 1 School of Chemical Engineering and Technology, Hebei University of Technology, Tianjin, 300130 China; 2 National-Local Joint Engineering Laboratory for Energy Conservation of Chemical Process Integration and Resources Utilization, Tianjin, China; 3 School of Environmental Science and Engineering, Tianjin University, Tianjin, 300072 China

**Keywords:** *Piriformospora indica*, *Medicago sativa*, Phytoremediation, Soil-contamination, Bioavailability, Rhizosphere

## Abstract

**Background:**

The coexistence of polycyclic aromatic hydrocarbons (PAHs) and heavy metals has deleterious effects on environmental quality. Few reports have studied the mechanisms of plant inoculation with *Piriformospora indica* to remediate PAH-metal co-contaminated soil by analyzing the chemical speciation of the contaminants. This study investigated the influence of the inoculation of *Medicago sativa* with *P. indica* to remediate soil co-contaminated with phenanthrene (a kind of PAH) and cadmium (a heavy metal) by analyzing plant growth, physiological parameters and chemical speciation in rhizosphere and nonrhizosphere soils.

**Results:**

The presence of *P. indica* significantly increased plant tolerance, chlorophyll *a*, chlorophyll *b*, maximum quantum efficiency of PSII photochemistry and electron transport rate values in phenanthrene- and/or cadmium-contaminated soil. *P. indica* inoculation in *M. sativa* roots increased fluorescein diacetate activities in soils contaminated with phenanthrene, cadmium or both, especially in the nonrhizosphere. The presence of phenanthrene prevented the inoculated plant from accumulating cadmium to some extent, whereas the presence of cadmium did not prevent the degradation of phenanthrene in either the rhizosphere or the nonrhizosphere after *P. indica* colonization. Although the low bioavailability of cadmium in the rhizosphere restricted its transportation into the stem, *P. indica* colonization in plants effectively increased cadmium accumulation in roots in soil co-contaminated with cadmium and phenanthrene.

**Conclusions:**

In conclusion, this work provides a theoretical basis for the use of *P. indica* combined with *M. sativa* for the remediation of PAH-metal co-contaminated soil.

## Background

The coexistence of heavy metals and polycyclic aromatic hydrocarbons (PAHs) in soil has caused environmental problems worldwide [1]. Heavy metals and PAHs are human health risks because of their cytotoxicity, mutagenicity and teratogenicity [2]. The co-contamination of heavy metals and PAHs leads to higher toxicity in the environment and increases the difficulty of remediating polluted soil. For example, PAH mineralization was inhibited by a high content of heavy metals in co-contaminated soil [3]. Phytoremediation has been regarded as a cost-effective and environmentally friendly technology and is widely used for soil remediation [4]. Plant-associated microorganisms significantly enhance the PAH and heavy metal removal efficiency of plants [5, 6]. Removal has two aspects: the fixation and isolation of PAHs and heavy metals by plant roots and the degradation of PAHs by microbes in the rhizosphere [7]. The presence of microorganisms protects plants against damage from plant pathogens and promotes plant growth; in turn, the presence of plants changes the microbial community structure and results in a more conducive environment for soil contaminant removal.

Arbuscular mycorrhizal fungi (AMF) have been used in the phytoremediation of soil contaminated with diesel [8] or heavy metals [9] to enhance plant resistance and heavy metal accumulation capacity. However, AMF are obligate endosymbionts that are unable to be cultivated in vitro. *P. indica* was first discovered in the rhizospheres of woody shrubs in the sandy desert soils of the Thar region of India [10]. The root endophytic basidiomycete *P. indica* belongs to the recently defined order Sebacinales [11]. Species of this order form a novel type of mutualistic mycorrhizal symbiosis and are able to colonize a broad spectrum of plants and convey various beneficial effects to the colonized host plants [12–15]. *P. indica* acts as a bioprotector against pathogens [16], alters plant secondary metabolites [17], increases nutrient uptake, promotes plant growth [18, 19], confers drought tolerance to Arabidopsis and barley [20, 21], and alleviates salt stress in barley and rice by increasing the activity of detoxifying enzymes as well as the photosynthetic pigment content in colonized plants [22, 23]; *P. indica* is different from AMF that can be cultivated in vitro*.* Considering the various beneficial effects of *P. indica,* this fungus is regarded as having significant agronomical and ecological application value [24]. However, there are few reports about the utilization of this endophyte for the remediation of soil contaminated by heavy metals and PAHs.

In the remediation of both heavy metals and PAHs, bioavailability determines the remediation efficiency [25, 26]. An increase in organic acid production by roots can effectively increase the bioavailability of heavy metals and PAHs [27, 28]. *P. indica* directly increases plant root biomass by producing indole-3-acetic acid (IAA) [29], which increases organic acid production in the roots. Similarly, biosurfactants produced by plant growth-promoting microorganisms (PGPM) can increase the bioavailability of heavy metals and PAHs [27, 28]. In terms of the remediation of heavy metals, the presence of heavy metals stimulated PGPM to produce siderophores, which in turn increased the bioavailability of heavy metals [30–32]. In the case of PAH remediation, plants provide an effective platform for recruiting more efficient microbes to digest or degrade the PAHs [1]. Therefore, PGPM combined with plant roots is an important method of remediating heavy metal- and PAH-contaminated soil.

Some researchers have reported using microorganisms to promote the remediation of single-source contamination caused by heavy metals or PAHs [33]; however, few studies have focused on the influence of plants inoculated with *P. indica* on the chemical speciation of heavy metals and PAHs in soils co-contaminated with metal and PAH. Notably, *P. indica* has been widely studied for its interaction with plants. However, this study focused on different aspects of this topic: (1) investigating the tolerance of *M. sativa* to phenanthrene and cadmium after *P. indica* colonization; (2) speculating on the rhizospheric effect of *P. indica* inoculation by measuring microbial activity and enzyme activity in the rhizosphere and nonrhizosphere; and (3) evaluating the effect of *P. indica* inoculation on the phytoremediation efficiency of *M. sativa* in phenanthrene-cadmium co-contaminated soil.

## Results

### *P. indica* inoculation increased the biomass of *M. sativa* in contaminated soil

**Fig. 1:**
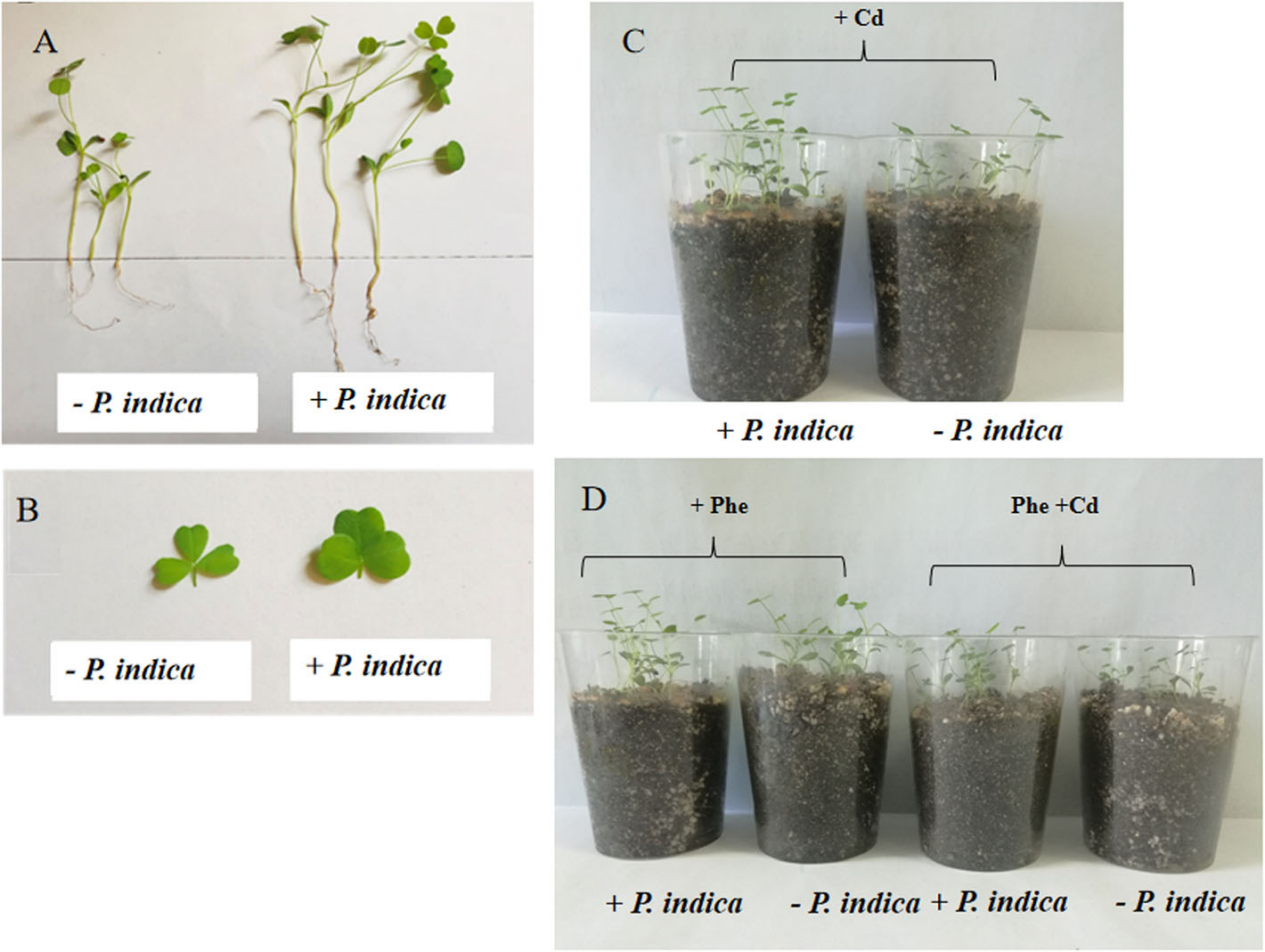
Biological effects on root and stem in the Phe, Cd and the combined Phe-Cd contaminated soil with or without *P. indica* inoculation. **a**: Control with or without *P. indica* inoculation. **b**: Leaves area were compared between *P. indica* inoculation and non-*P. indica* inoculation without any contamination. **c**: Above ground parts were compared between *P. indica* inoculation and non-*P. indica* inoculation in Cd contaminated soil. **d**: Above ground parts were compared between *P. indica* inoculation and non-*P. indica* inoculation in the Phe and the combined Phe-Cd contaminated soil

**Fig. 2:**
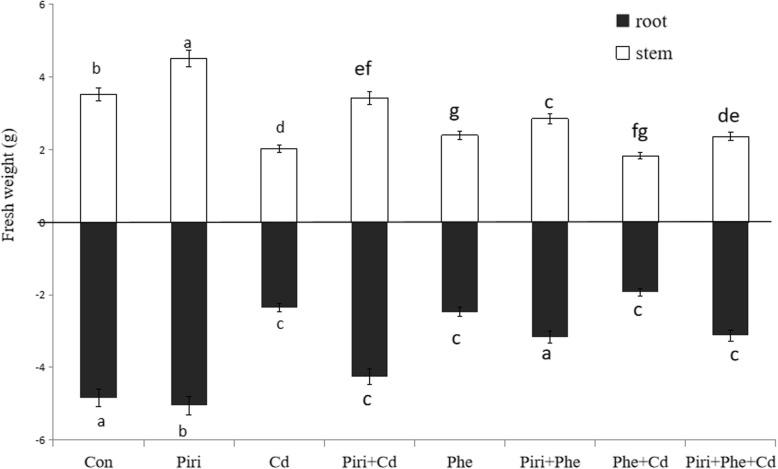
The statistical analysis on fresh weight of stems and roots in *M. sativa* were obtained in the control, Phe/Cd and the combined Phe-Cd contaminated soil with or without *P. indica* colonization. Control with (Piri) or without (Con) *P. indica* inoculation; Phe contaminated soil with (Piri+Phe) or without (Phe) *P. indica* inoculation; Cd contaminated soil with (Piri+Cd) or without (Cd) *P. indica* inoculation; Phe-Cd co-contaminated soil with (Piri+Cd + Phe) or without (Cd + Phe) *P. indica* inoculation. *M. sativa* was planted in all treatments. Each value of fresh weight is the mean of three replicates. Error bars show standard error. The fresh weight in different treatments was significantly reduced or increased compared to the control according to the Duncan’s Multiple Range Test (*P* < 0.05)

The biomass of *M. sativa* was recorded to reflect the tolerance of the plant to pollutants in soil. *P. indica* inoculation significantly promoted both root and stem biomass compared with those of the noninoculated plants (control) (Fig. 1a). In addition, the leaf area was increased compared to that in the control (Fig. 1b). However, reduced biomass (fresh weight) was observed in the Cd, Phe and Phe + Cd treatments without *P. indica* inoculation, whereas the addition of *P. indica* spores significantly increased the biomass in those treatments (Fig. 1c-d). The data from 1 hundred plants in each different treatment and the related statistical analysis are provided in Fig. 2. The Phe + Cd treatment led to the most serious biomass reduction compared with the control. In contrast, the plant biomass in the Piri+Cd, Piri+Phe and Piri+Phe + Cd treatments was higher than that in the corresponding treatments without *P. indica* inoculation. Notably, the growth inhibition in the Cd treatment was greater than that in the Phe treatment.

### Chlorophyll (Chl) a and b contents and fluorescence parameters

The differences in Chl *a* and Chl *b* contents between *P. indica* inoculation conditions were significant (*p* < 0.01) when plants were exposed to Cd and Phe contamination (Table 1). Without Cd or Phe contamination, *P. indica*-inoculated *M. sativa* plants had higher leaf Chl *a* and Chl *b* contents than the noninoculated plants. Both Cd and Phe contamination significantly reduced the Chl *a* and Chl *b* contents. The effects of Phe on the Chl *a* and Chl *b* contents were more severe than those of Cd. However, *P. indica* inoculation significantly increased the Chl *a* and Chl *b* contents under the Cd, Phe, and Cd + Phe treatments in comparison to those in the noninoculated plants. The presence of *P. indica* significantly reduced F0 so that the F0 values of the inoculated plants were 8, 12.9, and 14% lower than those of the noninoculated plants under the Cd, Phe, and Cd + Phe treatments, respectively. Additionally, all three fluorescence parameters, including Fm (maximal fluorescence level in the dark-adapted state), Fv/Fm (maximum quantum efficiency of PSII photochemistry), and ETR (relative PSII electron transport rate), were significantly decreased under the Cd, Phe, Cd + Phe treatments compared with those of the control. However, a noticeable increase in Fm, Fv/Fm, and ETR was observed in *P. indica*-inoculated *M. sativa* in comparison to noninoculated plants.

**Table 1:** The effects of *P. indica*, Cd and Phe treatments on Chl a, Chl b, F0, Fm, Fv/Fm and ETR in *M. sativa*

Fungal treatment	Phe and Cd treatment	Chl a (mg g^**− 1**^ FW)	Chl b (mg g^**− 1**^ FW)	F0	Fm	Fv/Fm	ETR
**-P**	**0**	**4.57** **+** **0.01**^**g**^	**2.63** **+** **0.02**^**a**^	**0.76** **+** **0.03**^**a**^	**2.78** **+** **0.03**	**0.74** **+** **0.07**^**b**^	**92.5** **+** **2.4**^**c**^
	**Cd** **Phe** **Cd + Phe**	**3.58** **+** **0.10**^**a**^	**2.10** **+** **0.05**^**a**^	**0.85** **+** **0.04**^**a**^	**2.66** **+** **0.04**^**a**^	**0.58** **+** **0.07**^**c**^	**88.5** **+** **1.12**^**b**^
		**3.21** **+** **0.02**^**b**^	**1.93** **+** **0.04**^**a**^	**0.93** **+** **0.01**^**b**^	**2.41** **+** **0.02**^**b**^	**0.45** **+** **0.06**^**d**^	**71.6** **+** **2.07**^**de**^
		**3.10** **+** **0.03**^**b**^	**1.76** **+** **0.02**^**b**^	**1.07** **+** **0.02**^**c**^	**2.17** **+** **0.03**^**c**^	**0.31** **+** **0.05**^**e**^	**67.5** **+** **3.02**^**a**^
**+P**	**0**	**5.01** **+** **0.02**^**c**^	**2.85** **+** **0.04**^**c**^	**0.63** **+** **0.05**^**d**^	**2.86** **+** **0.04**^**d**^	**0.86** **+** **0.02**^**f**^	**98.3** **+** **3.17**^**b**^
	**Cd**	**4.26** **+** **0.04**^**d**^	**2.55** **+** **0.06**^**d**^	**0.78** **+** **0.06**^**f**^	**2.72** **+** **0.06**^**e**^	**0.76** **+** **0.02**^**a**^	**104** **+** **2.45**^**c**^
	**Phe**	**4.02** **+** **0.02**^**e**^	**2.41** **+** **0.05**^**e**^	**0.81** **+** **0.03**^**e**^	**2.62** **+** **0.05**^**f**^	**0.61** **+** **0.03**^**b**^	**101.1** **+** **3.14**^**d**^
	**Cd + Phe**	**3.78** **+** **0.04**^**f**^	**2.17** **+** **0.03**^**f**^	**0.92** **+** **0.02**^**g**^	**2.31** **+** **0.03**^**g**^	**0.52** **+** **0.04c**	**93.8** **+** **2.53**^**e**^

− P: non-inoculation (control), + P: *P. indica.* Values are mean ± SE, *n* = 3. The same letter within each column indicates no significant difference among treatments using Duncan’s Multiple Range Test

### *P. indica* auxin production affected by phenanthrene and cadmium

**Fig. 3:**
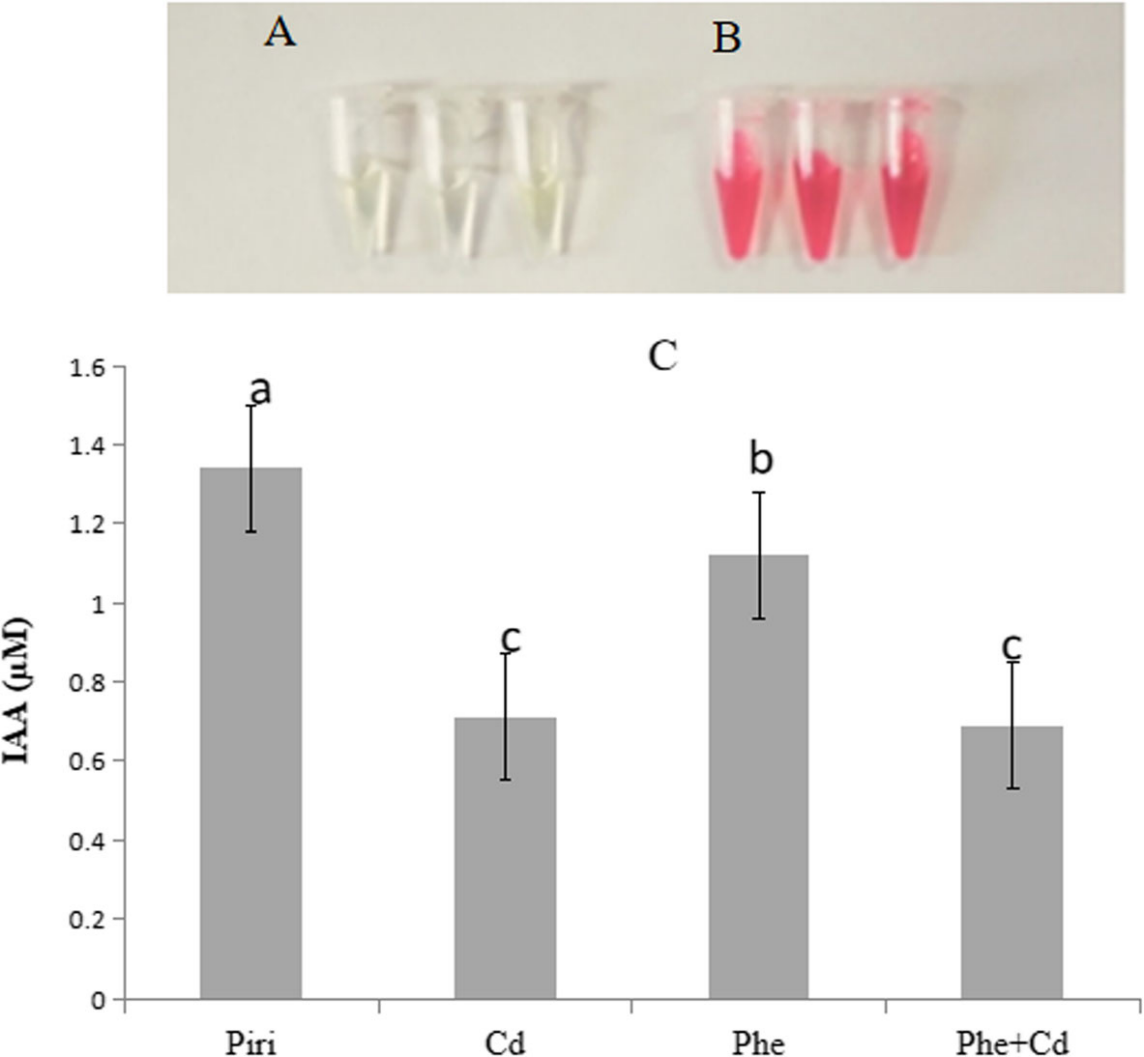
IAA production of *P.indica* during growth in CM medium containing the Cd or Phe contamination. Qualitative analysis by the Salkowski-method was performed to identify IAA production under phenanthrene, cadmium and the combined phenanthrene and cadmium treatments. **a**: After adding the tryptophan, the solution not containing *P. indica* spores under each treatment does not changed colour in each three repetitions. **b**: After adding the tryptophan, the solution containing *P. indica* spores under each treatment changed to pink colour in each three repetitions. **c**: IAA was determined in culture supernatants by HPLC-MS after 6 weeks containing the Cd, Phe or the combined Cd and Phe contamination medium, respectively. IAA concentrations in CM medium without any contamination were 1.34 + 0.03 μM (*n* = 3); IAA concentrations in the medium containing Cd were 0.71 + 0.05 μM (*n* = 3); IAA concentrations in the medium containing Phe were 1.12 + 0.07 μM (*n* = 3); IAA concentrations in the medium containing the combined Cd and Phe were 0.69 + 0.04 μM (*n* = 3); respectively. Error bars show standard error. The IAA concentration was significantly reduced in the Cd, Phe and the combined Cd + Phe treatments compared to the control (*P. indica*) according to the Duncan’s Multiple Range Test (*P* < 0.05)

IAA promotes plant growth at low concentrations and inhibits plant growth at high concentrations. Therefore, we were interested in revealing whether IAA production in *P. indica* would be affected by phenanthrene and cadmium. Qualitative analysis by the Salkowski method showed that after adding tryptophan, the solution containing spores of *P. indica* under each treatment changed to pink in three repetitions, unlike in the control (Fig. 3a-b), which indicated that IAA was produced under the Phe, Cd and combined Phe and Cd treatments. Additionally, the IAA content produced by *P. indica* was quantified by HPLC. The results showed that both the Phe and Cd treatments affected IAA production (Fig. 3c). Notably, the combined Phe and Cd treatment resulted in less IAA production (0.69 μM). This result explained why the biomass compensation in roots treated with combined Phe and Cd was less than that in roots treated with only Phe or Cd though all the roots in these treatments were colonized by *P. indica* (Fig. 1).

### *P. indica* inoculation increased FDA activities in nonrhizosphere soil

**Fig. 4:**
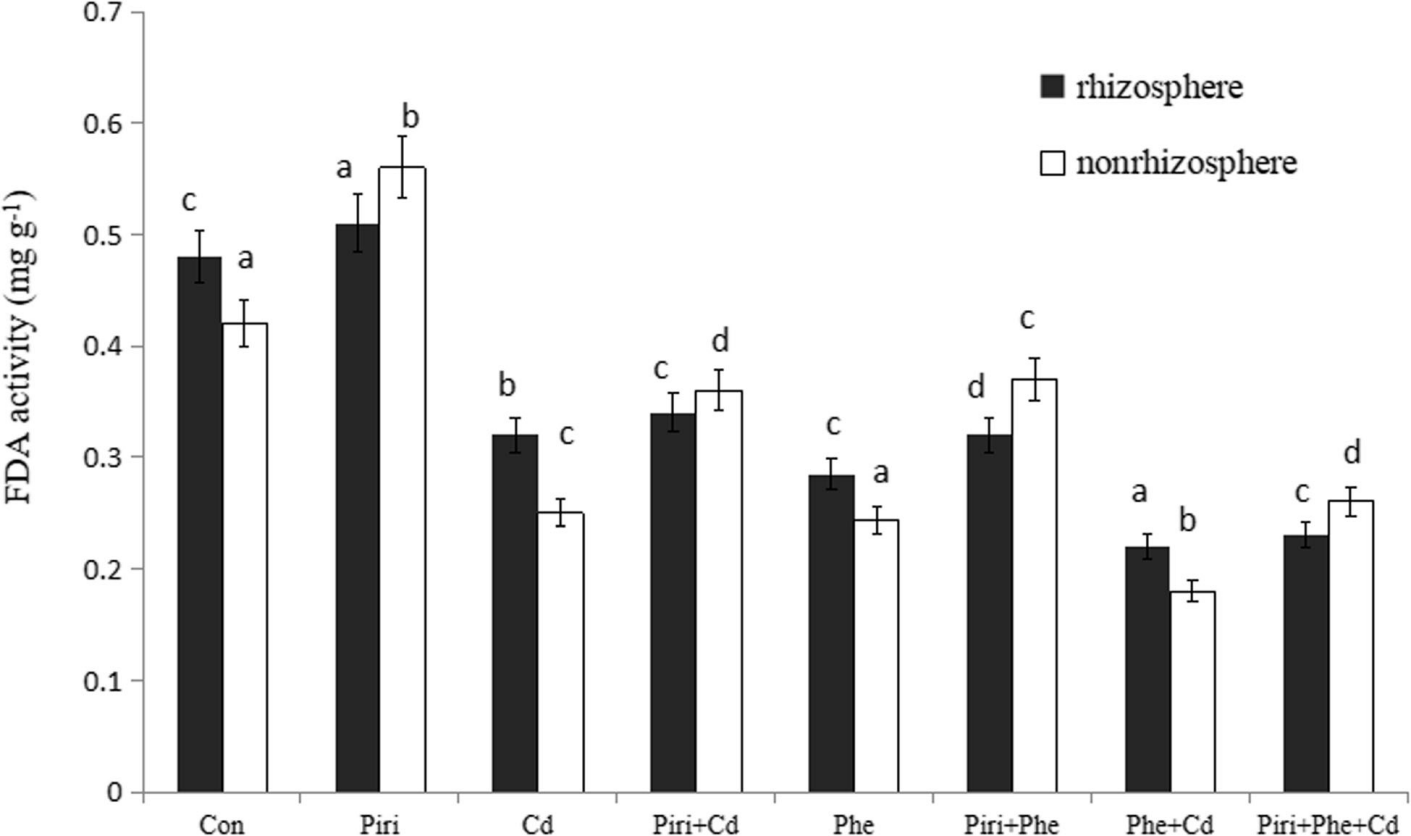
The FDA activities were identified in the control, Phe/Cd and the combined Phe-Cd contaminated soil with or without *P. indica* colonization. Control with (Piri) or without (Con) *P. indica* inoculation; Phe contaminated soil with (Piri+Phe) or without (Phe) *P. indica* inoculation; Cd contaminated soil with (Piri+Cd) or without (Cd) *P. indica* inoculation; Phe-Cd co-contaminated soil with (Piri+Cd + Phe) or without (Cd + Phe) *P. indica* inoculation. *M. sativa* was planted in all the treatments. Each value of FDA activities is the mean of three replicates. Error bars show standard error. For the control, Cd, Phe and the combined Cd + Phe treatments, FDA activity in the nonrhizosphere was significantly reduced compared with that in the rhizosphere; For the Piri, Piri+Cd, Piri+Phe and Piri+Cd + Phe treatments, FDA activity in nonrhizosphere was significantly increased compared with that in the rhizosphere; for Control and Piri, Cd and Piri+Cd, Phe and Piri+Phe, Phe + Cd and Piri+Phe + Cd, the adding of *P. indica* spores significantly increases the FDA activity both in the rhizosphere and nonrhizosphere compared with that in the treatment without *P. indica* inoculation. Different letters **a**-**d** in the columns indicate significant difference in FDA activities between treatments according to the Duncan’s Multiple Range Test (*P* < 0.05)

The fluorescein diacetate assay (FDA) is an easy and conclusive method for determining soil microorganism activity. Figure 4 shows that FDA activity was higher in *P. indica-*inoculated plants than in noninoculated plants in the combined and individual Cd and Phe treatments as well as in the control*.* The FDA activity in the nonrhizosphere under *P. indica* inoculation was the highest, whereas the Cd + Phe treatment in the nonrhizosphere had the lowest FDA activity. An interesting phenomenon was observed in which *P. indica* colonization in plant roots significantly increased microorganism activity in the nonrhizosphere compared with that in the rhizosphere. Cd and Phe contamination significantly reduced FDA activity both in the rhizosphere and the nonrhizosphere. In comparing the Cd and Piri+Cd treatments, no obvious differences in FDA activity were observed in the rhizosphere, and the addition of *P. indica* remarkably increased FDA activity in the nonrhizosphere. Similar results were obtained between the Phe and Piri+Phe treatments.

### *P. indica* inoculation increased polyphenol oxidase activity in the rhizosphere

**Fig. 5:**
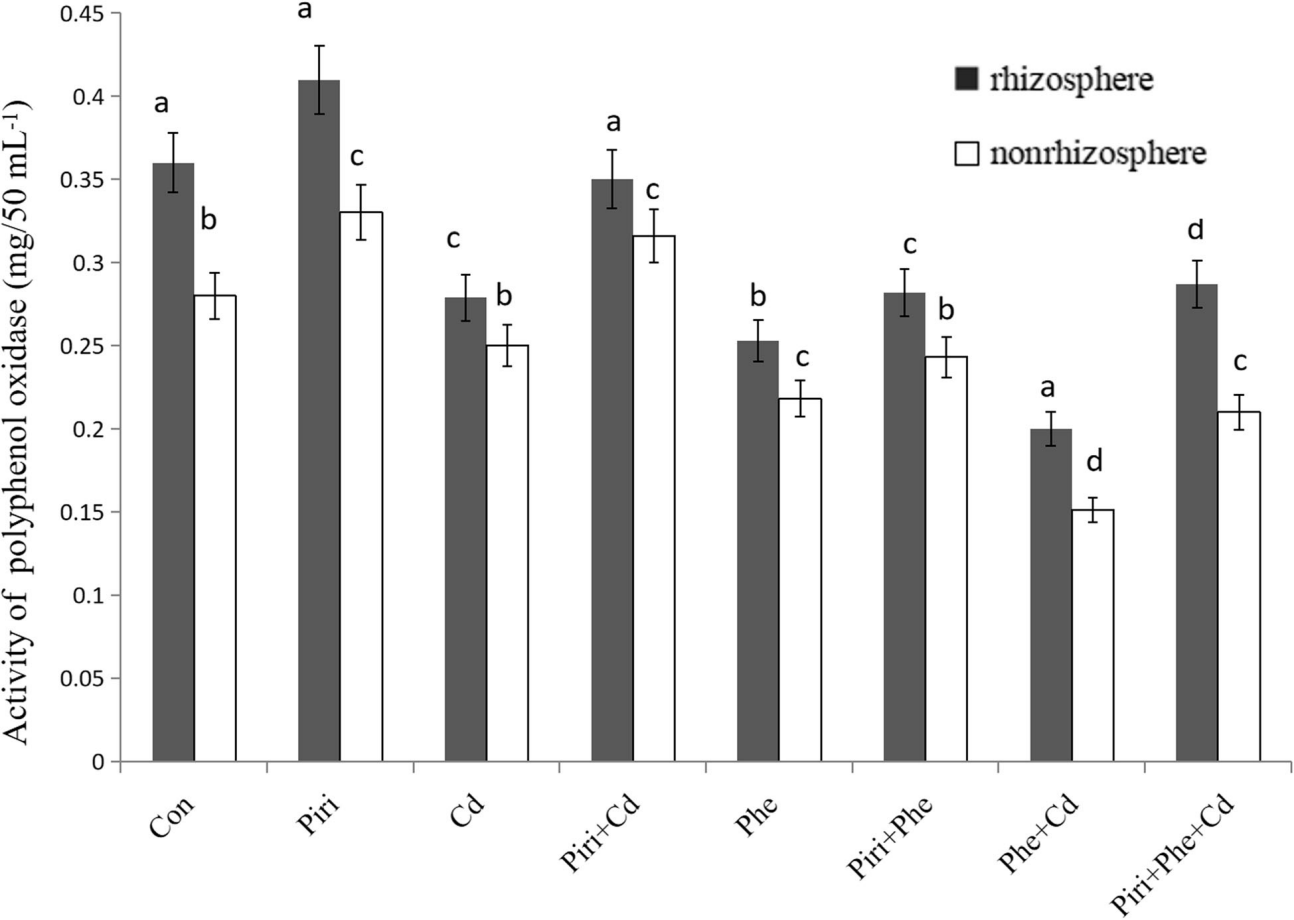
Polyphenol oxidase activity was identified in different treatments in the rhizosphere and nonrhizosphere Control with (Piri) or without (Control) *P. indica* inoculation; Phe contaminated soil with (Piri+Phe) or without (Phe) *P. indica* inoculation; Cd contaminated soil with (Piri+Cd) or without (Cd) *P. indica* inoculation; Phe-Cd co-contaminated soil with (Piri+Cd + Phe) or without (Cd + Phe) *P. indica* inoculation. *M. sativa* was planted in all treatments. Each value of fresh weight is the mean of three replicates. Error bars show standard error. For the Control and Piri, Cd and Piri+Cd, Phe and Piri+Phe, Phe + Cd and Piri+Phe + Cd, the adding of *P. indica* spores significantly increases the polyphenol oxidase activity both in the rhizosphere and nonrhizosphere compared with that in the treatment without *P. indica* inoculation. Different letters (a-d) in the columns indicate significant difference in polyphenol oxidase activity between treatments according to the Duncan’s Multiple Range Test (*P* < 0.05)

Polyphenol oxidase activity in the rhizosphere and nonrhizosphere is shown in Fig. 5. Polyphenol oxidase activity in the rhizosphere was higher than that in the nonrhizosphere. *P. indica* colonization significantly increased polyphenol oxidase activity in both the rhizosphere and the nonrhizosphere*.* The order of polyphenol oxidase activity from low to high among the different treatments was Phe + Cd < Phe < Cd < Piri+Phe < Piri+Phe + Cd < Piri+Cd < Con < Piri. These data revealed several findings: 1) polyphenol oxidase activity in soil was more easily affected by Phe than by Cd contamination; 2) the addition of *P. indica* significantly increased polyphenol oxidase activity in the rhizosphere; 3) in Phe and Cd co-contaminated soil, the polyphenol oxidase activity was greatly inhibited, whereas after *P. indica* colonization in the *M. sativa* roots, the polyphenol oxidase activity was remarkably enhanced compared with that in the control.

### *P. indica* inoculation reduced phenanthrene content in the rhizosphere

**Fig. 6:**
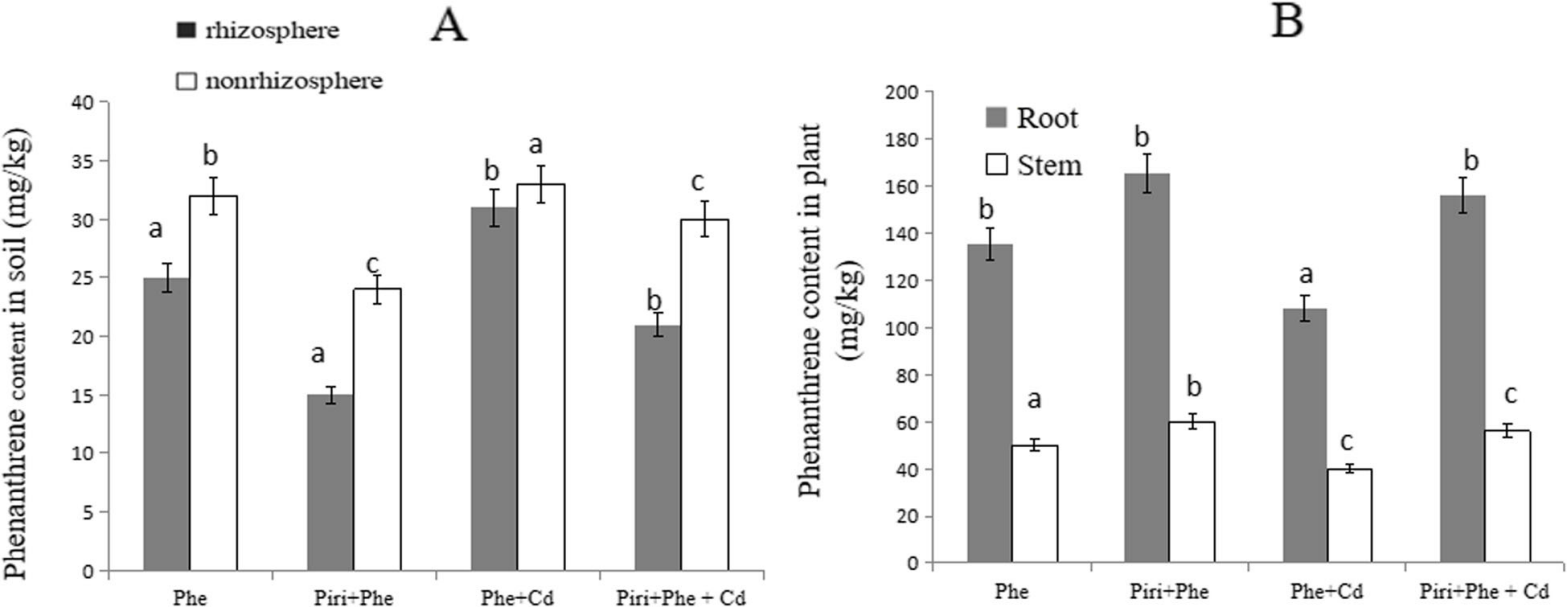
Phenanthrene content was identified in soil and plant. **a**: The phenanthrene content was identified in different treatments in the rhizosphere and nonrhizosphere. **b**: The phenanthrene content was identified in different treatments in root and stem. The Phe contaminated soil with (Piri+Phe) or without (Phe) *P. indica* inoculation; the Phe-Cd co-contaminated soil with (Piri+Cd + Phe) or without (Cd + Phe) *P. indica* inoculation. *M. sativa* was planted in all treatments. Each value of phenanthrene content is the mean of three replicates. Error bars show standard error. For the Phe and Piri+Phe, Cd + Phe and Piri+Cd + Phe, the adding of *P. indica* spores significantly reduced/increased phenanthrene content in the rhizosphere and nonrhizosphere/plant (root and stem) compared with that in the treatment without *P. indica* inoculation. Different letters **a-c** in the columns indicate significant difference in phenanthrene content between treatments according to the Duncan’s Multiple Range Test (*P* < 0.05)

The phenanthrene degradation rates in the rhizosphere and nonrhizosphere were measured (Fig. 6a). Generally, the phenanthrene content in the rhizosphere was lower than that in the nonrhizosphere. In the rhizosphere, among the four different treatments, the phenanthrene concentration in the soil from high to low was Phe + Cd, Phe, Piri+Phe + Cd, and Piri+Phe. The concentration gradient in the nonrhizosphere was similar to that in the rhizosphere. These data imply that *P. indica* colonization effectively reduced phenanthrene content in soil.

To support this evidence, the phenanthrene concentrations in plant roots and stems were also measured. The phenanthrene concentrations in roots were 135 ± 6 mg kg^− 1^, 165 ± 4 mg kg^− 1^, 108 ± 10 mg kg^− 1^, and 156 ± 7 mg kg^− 1^ among the Phe, Piri+Phe, Phe + Cd and Piri+Phe + Cd treatments, respectively (Fig. 6b). Notably, the phenanthrene concentrations in roots were higher than those in stems. The Phe + Cd treatment had the lowest phenanthrene content in the stem compared to the other treatments. There was no striking difference in the stem phenanthrene content among the Phe, Piri+Phe and Piri+Phe + Cd treatments.

### *P. indica* inoculation reduced cadmium content in the rhizosphere

**Fig. 7:**
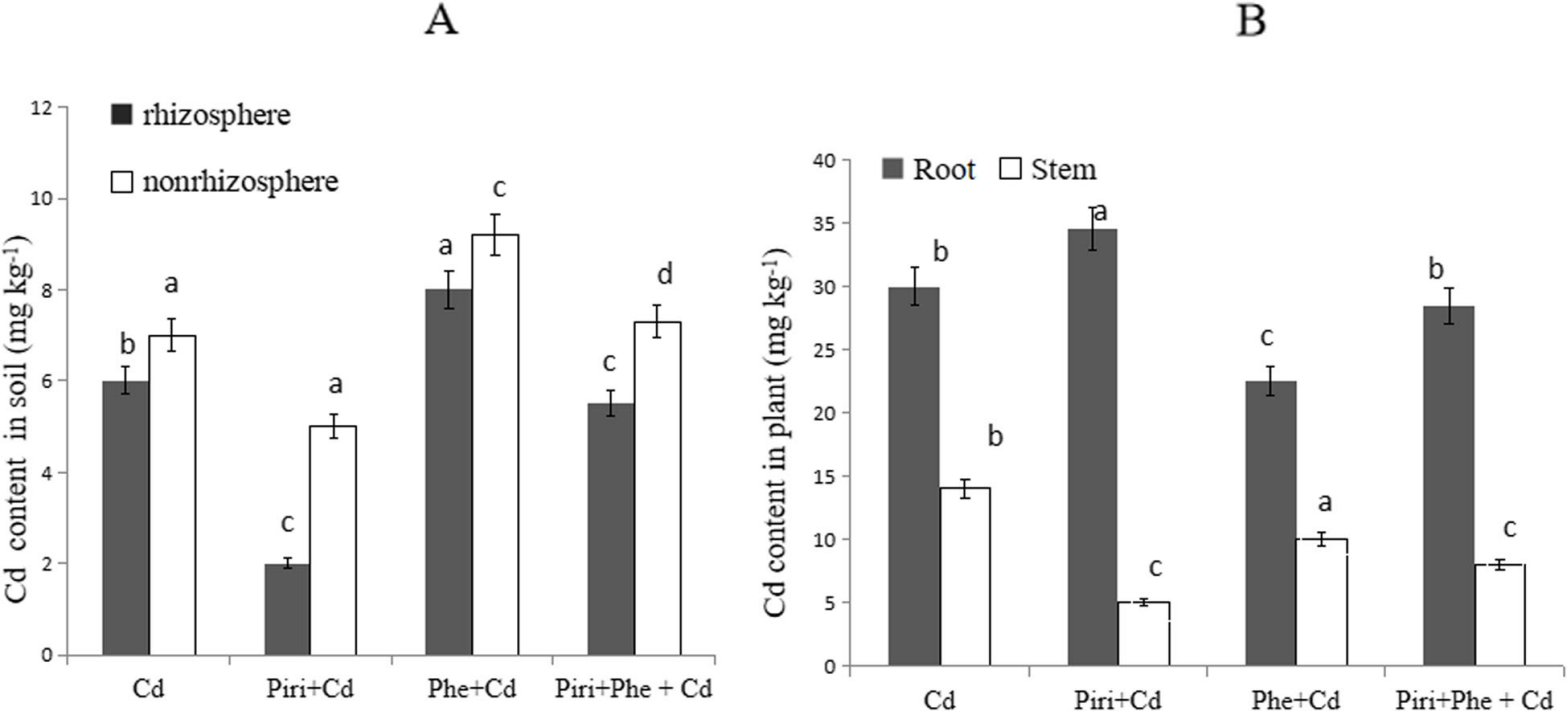
The cadmium content was identified in soil and plant. **a**: The cadmium content was identified in different treatments in the rhizosphere and nonrhizosphere. **b**: The cadmium content was identified in different treatments in root and stem. Cd contaminated soil with (Piri+Cd) or without (Cd) *P. indica* inoculation; Phe-Cd co-contaminated soil with (Piri+Cd + Phe) or without (Cd + Phe) *P. indica* inoculation. *M. sativa* was planted in all treatments. Each value of cadmium content is the mean of three replicates. Error bars show standard error. For Cd and Piri+Cd, Cd + Phe and Piri+Cd + Phe, the adding of *P. indica* spores significantly reduced/increased the cadmium content in the rhizosphere and the nonrhizosphere/plant (root and stem) compared with those in the treatments without *P. indica* inoculation. Different letters **a-c** in the columns indicate significant difference in the cadmium content between treatments according to the Duncan’s Multiple Range Test (*P* < 0.05)

The cadmium content in the rhizosphere and nonrhizosphere was measured and is shown in Fig. 7a. Similarly, the cadmium content in the rhizosphere was less than that in the nonrhizosphere. In the rhizosphere, among the four different treatments, the cadmium concentration from high to low was Phe + Cd, Piri+Phe + Cd, Cd, and Piri+Cd. Adding *P. indica* spores significantly reduced the cadmium content in comparison to that in the treatment without *P. indica* inoculation. For instance, in both the rhizosphere and the nonrhizosphere, the Piri+Cd treatment had a lower cadmium content than the Cd treatment, and the Piri+Phe + Cd treatment had a lower cadmium content than the Phe + Cd treatment. Remarkably, the Cd content in the rhizosphere of the *P. indica* treatment was significantly lower (less than 2 mg kg^− 1^) than the original Cd content in the soil (10 mg kg^− 1^). The cadmium concentration gradient in the nonrhizosphere was similar to that in the rhizosphere. These data imply that colonization with *P. indica* effectively reduced the cadmium content in soil.

Additionally, the cadmium concentrations in plant roots and stems were explored (Fig. 7b). The cadmium concentrations in roots were 30 ± 4 mg kg^− 1^, 34 ± 6 mg kg^− 1^, 23 ± 8 mg kg^− 1^, and 29 ± 5 mg kg^− 1^ in the Cd, Piri+Cd, Phe + Cd and Piri+Phe + Cd treatments, respectively. Likewise, the cadmium concentrations in roots were notably higher than those in stems. In stems, the Cd content showed a different trend from the phenanthrene content: the Piri+Cd treatment had a lower Cd content than the Cd treatment, and the Cd content in the Piri+Phe + Cd treatment was lower than that in the Phe + Cd treatment. This result suggested that inoculation with *P. indica* inhibited the transport of Cd into the stem.

## Discussion

Heavy metal and organic pollutants in soil severely harm plant growth and development. Cd treatment substantially inhibits plant biomass accumulation. Shahabivand et al. [34] reported that *P. indica* successfully colonized plants grown in 120 mg kg^− 1^ Cd-contaminated soil, which suggests that this fungus was able to colonize roots under high Cd stress in soil. However, we found that the spore germination and hyphal growth of *P. indica* were seriously inhibited under 40 mg kg^− 1^ Cd (data not shown). Therefore, 10 mg kg^− 1^ Cd was selected as the test concentration.

Our data indicated that colonization with *P. indica* in the Cd (10 mg kg^− 1^), Phe (40 mg kg^− 1^) and Phe + Cd treatments relieved the stress from heavy metals and PAHs. Though the Cd concentration in the roots of the Piri+Cd treatment was higher than that in the Cd treatment, the biomass was not significantly affected. The results implied that a high concentration of Cd could be fixed or isolated to some degree by this fungus and plant co-living system which would therefore reduce the harmful effects to the plants. Cd accumulation in stems was reduced after phenanthrene contamination in soil, which indicated that the organic pollutants accumulated in roots might hinder Cd transport from roots to aboveground parts. The Piri+Phe treatment accumulated a high concentration of phenanthrene in the roots, which decreased the biomass of the roots and stems compared to that in the Piri treatment. However, even the high concentration of phenanthrene in roots did not hinder the growth promotion effect of *P. indica* on host plants compared to the growth of plants in the Phe treatment. This result was different from a previous report that the accumulation of pyrene by plant-growth-promoting bacteria (PGPB) decreased stem biomass [32]. One possible reason for this different result is that the endophytic fungus, unlike the bacteria, could accumulate and tolerate the phenanthrene, thereby reducing its toxicity to plants.

*M. sativa* plants subjected to Cd and Phe stress showed reduced growth in terms of shoot and root length and shoot and root fresh weight. The toxic effects of Cd have widely been reported in different plant species, and Cd is known to reduce or inhibit plant growth because of the harmful impact of Cd on the processes of photosynthesis, respiration and essential element uptake [34, 35]. Chaoui and Ferjani (2005) [36] reported that the activity of indole-3-acetic acid (IAA) oxidase (as a growth-limiting enzyme) was increased under Cd toxicity, which resulted in reduced plant growth due to the diminution of the endogenous content of the plant growth-promoting hormone auxin. In our work, the elevation in host photosynthetic efficiency via increased Chl *a* and *b* contents and elevated Fv/Fm and ETR values (Table 1) contributed to the *P. indica*-induced growth promotion and stress tolerance.

Soil microorganism activity can effectively reflect soil fertility. The soil microorganism activity was determined in order to speculate on phytoremediation efficiency. We noticed that the FDA activity in the nonrhizosphere was higher than that in the rhizosphere in the *P. indica*-colonized plants. One possible reason for this result is that, due to the presence of *P. indica* in the plant roots, much more heavy metal accumulated in the rhizosphere than in the nonrhizosphere, which resulted in harmful effects on microorganisms and therefore reduced FDA activity in the rhizosphere. The FDA activity in the *P. indica* treatment was higher than that in the non-*P. indica* treatment in both the rhizosphere and the nonrhizosphere. The Phe treatment had lower FDA activity than the Cd treatment, which was in accordance with a previous report that petroleum hydrocarbons inhibit microorganism activity in soil [37]. Though similar FDA activities were observed in the rhizosphere in the Cd and Piri+Cd or Phe and Piri+Phe treatments, the mechanisms of FDA activity might be different. The reduction in FDA activity in the Cd and Phe treatments was probably due to heavy metal or PAH stress, whereas the reduction in FDA activity in the Piri+Cd or Piri+Phe treatments was probably due to the inhibition of phytopathogen growth. Enzymes are part of the soil composition, and the degree of their activity sensitively reflects the direction and strength of biochemical reactions in soil [38]. The changes in enzyme activity effectively reflect the ability of microorganisms and plant roots to degrade organic pollutants in soil. In our experiment, we detected the activity of several enzymes, including urease and invertase, and the results indicated that these enzymes were more active in the rhizosphere of *P. indica*-colonized roots than in the rhizosphere of noninoculated plants (data not shown). Polyphenol oxidase is an important oxidoreductase enzyme in soil that participates in the process of decomposition and transformation of aromatic compounds. The enhanced polyphenol oxidase activity in this study implied that *P. indica* has a strong ability to remediate contaminated soil.

Co-contamination with heavy metals and PAHs resulted in a deleterious influence on microorganism activity in soil compared to that under individual heavy metal or PAH contamination [39], which explained why the Phe + Cd treatment had the lowest FDA activity in all treatments. The Piri+Cd and Piri+Phe + Cd treatments had higher FDA activity than the Cd and Phe + Cd treatments, respectively, which implied that the endophyte and plant co-living system contributed to a well-established microecosystem for microorganisms in soil.

In the Phe, Piri+Phe, Cd + Phe and Piri+Phe + Cd treatments, Phe accumulation in plants was 135 + 6 mg kg^− 1^, 165 + 4 mg kg^− 1^, 108 + 10 mg kg^− 1^, and 156 + 7 mg kg^− 1^, respectively. The high concentration of Phe in plants means that less Phe remained in the soil. In addition to plants, soil microorganisms play key roles in the removal of PAHs. In the Phe + Cd treatment, the Phe concentrations remaining in the rhizosphere and nonrhizosphere were higher than those in the Phe treatment, which was reasonable due to the higher microorganism activity in the Phe treatment. Though no significant difference in FDA activity was detected between the Phe and Phe + Piri treatments, *P. indica* colonization obviously reduced the Phe concentration in the rhizosphere and the nonrhizosphere. Similar data were collected from the Phe + Cd and Phe + Cd + Piri treatments. In total, the Phe concentration in the rhizosphere was lower than that in the nonrhizosphere, suggesting that the rhizosphere effect was responsible for the lower accumulation of PAHs in the rhizosphere soil than in the nonrhizosphere soil [40].

The order of Cd concentrations in roots from high to low was as follows: Piri+Cd, Cd, Piri+Phe + Cd, and Phe + Cd. The data indicated that *P. indica* colonization increased Cd accumulation in roots. However, in comparing the Piri+Cd and Piri+Phe + Cd treatments, it was notable that the addition of Phe reduced Cd accumulation into a certain degree. The bioavailability of heavy metals determines phytoremediation efficiency [41]. Microorganisms in soil, such as fungi and bacteria, are capable of producing biosurfactants such as rhamnolipids that can increase the mobility of heavy metals to enhance their bioavailability [42]. Therefore, it was reasonable that *P. indica* colonization significantly reduced the Cd concentration in the rhizosphere compared with that in the nonrhizosphere. On the other hand, plant root exudates also contribute to the acidification of heavy metals, which increases the efficiency of phytoremediation. *P. indica* colonization significantly promoted root growth and development, and the roots in turn secreted exudates to increase the bioavailability of heavy metals and enhance phytoremediation efficiency in the rhizosphere, forming a virtuous cycle.

## Conclusions

**Fig. 8:**
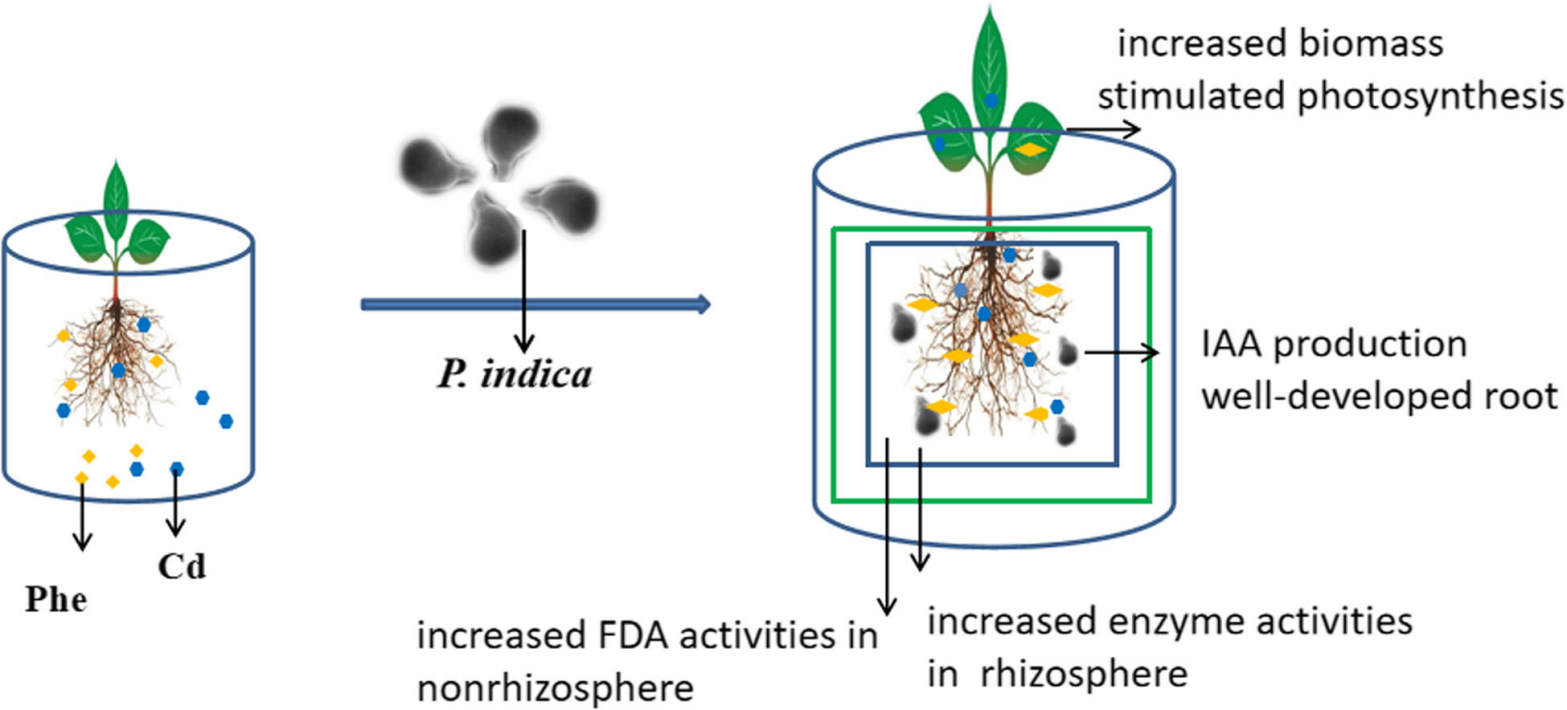
The phytoremediation effect of *M. sativa* colonized by *P. indica* in phenanthrene and cadmium co-contaminated soil. Four benefits aspects: including increased biomass, well-developed root, increased FDA activities in the rhizosphere and nonrhizosphere as well as increased enzyme activities in the rhizosphere were obtained after *P. indica* colonization in *M. sativa* root under phenanthrene and cadmium co-contaminated soil

In conclusion, *P. indica* colonization had positive effects on photosynthetic processes and increased the tolerance of *M. sativa* in soil contaminated with phenanthrene, cadmium, and combined cadmium and phenanthrene, especially in soil only contaminated with cadmium. The phenanthrene and cadmium co-contaminated soil affected soil microorganisms more severely than individual phenanthrene or cadmium contamination. *P. indica* inoculation in *M. sativa* roots increased the FDA activity in soils contaminated with phenanthrene, cadmium and both pollutants, especially in the nonrhizosphere. The addition of *P. indica* stimulated the roots of *M. sativa* to accumulate higher concentrations of phenanthrene and cadmium, thereby enhancing phytoremediation efficiency. A clear diagram of our conclusions about the phytoremediation effect of *M. sativa* colonized by *P. indica* on soil co-contaminated with phenanthrene and cadmium is provided in Fig. 8. This study suggests that the application of *P. indica* combined with *M. sativa* could contribute to the remediation of PAH-metal co-contaminated soil for sustainable agriculture.

## Methods

### Plant materials

*Medicago sativa* seeds were provided by Professor Diter Von Wettstein, Department of Crop and Soil Sciences, Washington State University.

### Chemicals

Phenanthrene with a purity of 98% was purchased from Sigma (USA). The rest of the chemicals were purchased from Dingguo (Tian Jin, China).

### Soil used in the study

The experimental soil had never been contaminated by PAHs or heavy metals and was collected from the topsoil (0–20 cm) at Hebei University of Technology, China. The soil is a phaeozem (alfisol) from Hebei Province [43]. The test soil was sieved with a 2 mm sieve after being air-dried. The soil samples were measured using standard methods [44] before phytoremediation. The composition of the sample soil was physico-chemically characterized: 55.6 ± 2.1% silt, 30.8 ± 1.8% sand and 13.6 ± 1.5% clay; 0.06% total N, 8 mg/kg available P, 40 mg/kg available K, and 1.1% organic matter. The soil pH was 7.36 ± 0.06.

### Preparation of phenanthrene-contaminated soil

Phenanthrene was dissolved in acetone and added to a small part of the soil. One day later, after the acetone had volatilized, a small part of the soil was added to the whole sample soil and incorporated thoroughly. The final concentration of phenanthrene in the soil was measured as 40 ± 3 mg kg^− 1^.

### Preparation of cadmium-contaminated soil

An aqueous solution of cadmium nitrate was added to the prepared soil, and the final concentration of cadmium in the soil was measured as 10 ± 2 mg kg^− 1^.

### Preparation of phenanthrene and cadmium co-contaminated soil

The acetone stock solution containing phenanthrene was first added to the test soil. After the acetone evaporated, the cadmium nitrate aqueous solution was added to the soil previously polluted by phenanthrene. The final concentrations of cadmium and phenanthrene in the soil were measured as 10 ± 2 mg kg^− 1^ and 40 ± 3 mg kg^− 1^, respectively. The prepared soil and a control soil without any pollution were moved into boxes and aged in the dark for 15 days.

### Fungal culture

In this work, the *P. indica* isolate DSM11827 (German collection of microorganisms and cell cultures in Braunschweig, Germany) was applied. The *P. indica* was supplied by Karl-Heinz-Kogel (Institute of Plant Pathology and Applied Zoology, Giessen, Germany). The *P. indica* was maintained at 23 °C on CM medium [45]. For solid medium, 14 g L^− 1^ agar was added; for liquid cultures, 100 mL medium was inoculated in a 300-mL Erlenmeyer flask. To test whether Cd and Phe would affect IAA production, 5 mg kg^− 1^ and 20 mg kg^− 1^ cadmium and phenanthrene, respectively, were added to the liquid cultures. The CM medium was inoculated with 20 mycelium plugs from the margin of a growing colony of *P. indica* on CM solid medium. The liquid cultures were incubated at 23 °C at 150 rpm on a rotary shaker.

### Quantification of IAA in fungal growth media by HPLC

The IAA production ability of *P. indica* under the Phe, Cd and Phe + Cd treatments was measured using the Salkowski method according to the literature [46]. The quantification of IAA in the fungal growth media was performed by HPLC. Fungal culture filtrates were harvested, acidified and extracted twice with ethyl acetate as described in [29]. The parameters used for HPLC were as follows: 50% methanol: 45% water: 5% acetonitrile (v/v) was used as the mobile phase. A flow rate of 0.2 mL min^− 1^ was applied. The injection volume was 10 μl. The column temperature was kept at 40 °C.The IAA content was quantified by Agilent HPLC equipped with an HC^R^ C18 (5 μm, 4.6 × 250 mm, Agilent, USA) reverse-phase chromatographic column. The IAA concentrations were always determined in parallel in medium in which no fungus had been cultured but which had been incubated under the same conditions.

### *M. sativa* treatment and *P. indica* inoculation

*P. indica* grown on CM medium plates for 3–4 weeks was used for the preparation of the spore suspensions. To collect spores from the CM agar plates, sterilized water containing 0.05% Tween-20 was added. The spores were released by gently scratching the surface of plates with a spatula, and the suspension solution was filtered through Miracloth (Calbiochem, Bad Soden, Germany) to remove the mycelium. After that, spores were collected by centrifuging the suspension solution at 3500 rpm for 7 min. Then, the spores were washed at least 3 times with sterilized Tween-H2O. By using a hemacytometer in combination with a microscope, the spore densities were determined. The spore concentration was adjusted to 500,000 spores/mL with sterilized Tween-H2O. For inoculation, 3 mL spore suspension was pipetted on top of plant roots in a square Petri dish. The seeds of *M. sativa* were surface-sterilized in 70% alcohol for 1 min and then in 3% NaClO for 15 min. After sterilization, seeds were repeatedly washed with sterile deionized water and planted in the MS medium [47] for germination. After 7 days, when roots had grown, *P. indica* spores were added to the root surface. Then, the seedlings containing *P. indica* spores were transferred into the different types of soil in pots (5 kg). Three replicates were applied for each treatment.

The plants were rejuvenated in the shade for 1 week. Then, the pots were transferred to a greenhouse with natural light and watered daily to maintain soil moisture (approximately 300 mL water/pot). Three months later, the plants were harvested, and soil from the rhizosphere and nonrhizosphere of *M. sativa* was collected*.*

### Measurement of chlorophyll contents and chlorophyll fluorescence

Chlorophyll content in the youngest fully expanded leaves (0.1 g) was extracted by 80% acetone and centrifuged at 4000 rpm for 20 min; the optical density of the supernatant was read at 663 and 645 nm wavelengths for Chl *a* and Chl *b*, respectively [48]. The following parameters of chlorophyll fluorescence were measured by analyzing the first fully grown leaves of *M. sativa* using a portable fluorometer (Hansatech, Instruments LTD, UK): F0 (minimal fluorescence level in the dark-adapted state), Fm (maximal fluorescence level in the dark-adapted state), Fv/Fm (maximum quantum efficiency of PSII photochemistry) and ETR (the relative PSII electron transport rate). *M. sativa* plants were dark-adapted for 30 min to measure the influence of the experimental factors on photosystem II (PSII) efficiency.

### Measure of soil microorganism activity

Soil microorganism activity was measured according to methods in the literature [31]. First, the soil was freeze-dried by a freeze-drying machine (Alpha 1–2 L D plus, Germany). Five grams of freeze-dried soil was dissolved in 15 mL of phosphate buffer solution (NaCl–8.5 g, Na_2_HPO_4_–2.2 g, NaH_2_PO_4_–0.1 g, pH 7.6) at room temperature. The turbid liquid was shaken at 180 rpm for 30 min, and then 0.5 mL of fluorescein diacetate (FDA) (2 g L^− 1^, in acetone) solution was added into the mixture. The absorbance value was recorded at OD_490nm_.

### Analysis of phenanthrene in the rhizosphere and nonrhizosphere

A three-step sequential extraction method was used to detect the concentrations of phenanthrene in different chemical speciations in the soil [40]. The ultrasonic extraction and high-performance liquid chromatography (HPLC) ultraviolet detection method was used. Ten grams of freeze-dried soil sample was dissolved in 50 mL of acetone-hexane (1:1, v/v) mixed extraction solvent. The extraction process was performed for 1 h in the ultrasonic cleaner. The extracted liquid was poured into a filter funnel containing 10 g anhydrous Na_2_SO_4_. The extracted liquid was concentrated to 5 mL by a rotary evaporation instrument in a 60 °C water bath. Then, 5 mL concentrated extracted liquid was transferred to a silica gel-alumina column for chromatography and eluted with a methylene chloride-hexane (1:1, v/v) mixture. The condensed elution was nearly dried, diluted to a final volume of 1 mL and used for HPLC determination. The parameters used for HPLC were as follows: methanol and water (87:13, v/v) were used as the mobile phase. A flow rate of 1 mL min ^– 1^ and detection wavelength of 254 nm were applied. The content of phenanthrene was quantified by Agilent HPLC equipped with an HC^R^ C18 (5 μm, 4.6 × 250 mm, Agilent, USA) reverse-phase chromatographic column. The phenanthrene recovery from soil was 97 ± 3%.

### Analysis of phenanthrene in plants

The phenanthrene in plants was extracted by acetone and dichloromethane (v/v, 1:1). After centrifugation and rotary evaporation, the concentrated phenanthrene was exchanged with 1 mL hexane for analysis. The content of phenanthrene was quantified by HPLC equipped with an HC^R^ C18 (5 μm, 4.6 × 250 mm, Agilent, USA) reverse-phase chromatographic column. The oven temperature was first maintained at 100 °C for 2 min, then increased to 300 °C at a rate 10 °C min^− 1^, and finally kept at 300 °C for 10 min. A phenanthrene standard was added to the uncontaminated plants and soil to measure phenanthrene recovery. A procedural blank as well as a spiked blank and duplicate samples were included every batch of 10 samples in the analysis. The phenanthrene recovery from plants was 99 ± 5%.

### Analysis of cadmium in soil and plants

Cadmium was determined according to methods in the references [40, 49]. Soil from the rhizosphere and nonrhizosphere of *M. sativa* was extracted by mixing 0.5 g soil with 10 mL HCl solution and then heating for 3 h (45 °C). After cooling, cadmium was extracted by mixing the soil with HNO_3_ and HClO_4_ (v/v, 4:1) for digestion (220 °C, 1 h) and then adding HF and HClO_4_ (v/v, 5:1) for further digestion (220 °C, 2 h). The same method was used to extract cadmium from the plants. At the end of the extraction step, the supernatant was harvested by centrifuging at 6000 rpm for 20 min. Then, the supernatant was filtered through a 0.45 μm microfiltration membrane and quantified by inductively coupled plasma optical emission spectrometry (ICPOES).

### Analysis of enzyme activity in the rhizosphere and nonrhizosphere

The enzyme activity of polyphenol oxidase was determined according to the soil enzyme analytical methods manual [50]. Ten grams of freeze-dried soil was dissolved in 10 mL pyrogallol (1%) and shaken at 150 rpm min^− 1^ for 1 min. Then, the sample was kept in the dark at 30 °C for 2 h. Four milliliters of citric acid-phosphate buffer (disodium hydrogen phosphate-35.61 g L^− 1^, citric acid-21.01 g L^− 1^, pH 4.5) was added to the sample. Finally, 35 mL ether was added and shaken for 2 min. The absorbance value at 430 nm was recorded after 30 min of extraction.

### Statistical analysis

In this study, all data are expressed as the means ± SE and represent at least three independent biological experiments. The significance of differences was analyzed by using one-way analysis of variance (ANOVA) with Duncan’s multiple range test.

## Data Availability

The datasets used and/or analyzed during the current study are available. from the corresponding author on reasonable request.

## Abbreviations

AMF: Arbuscular mycorrhizal fungi
Cd: cadmium
Chl: *a/* Chl *bChlorophyll (Chl) a, b*
ETR: The relative PSII electron transport rate
F0: Minimal fluorescence level in the dark-adapted state
FDA: Fluorescein diacetate assay
Fm: Maximal fluorescence level in the dark-adapted state
Fv/Fm: Maximum quantum efficiency of PSII photochemistry
IAA: Indole-3-acetic acid
*M. sativa*: *Medicago sativa*
PAHs: Polycyclic aromatic hydrocarbons
*P. indica*: *Piriformospora indica*
PGPM: Plant growth promoting microorganism
Phe: Phenanthrene
